# Supplementary material for: Phage SPO1 Protein Gp49 Is a Novel RNA Binding Protein That Is Involved in Host Iron Metabolism
Source: Int J Mol Sci. 2023 Sep 20;24(18):14318. doi: 10.3390/ijms241814318 (PMC10531801; doi:10.3390/ijms241814318)
Supplement: Supplementary file 1 [file ijms-24-14318-s001.zip › Sup_tables1-3/Table S1.pdf]

Table S1: Oligos used in the study (5’-3’)

DNA1	TGCTTATCAATTTGTTGCACC
DNA2	GGTGCAACAAATTGATAAGCA
ssRNA	AUGAAUAGUUAAACAACGUGG
pHT01_F	AAGGAGGAAGGATCCATGATTAAAGCGGCTG
pHT01_R	GACGTCCCCGGGGCATTACAGATTGATCCAATGA
pET_F	ATGATTAAAGCGGCTG
pET_R	TTACAGATTGATCCAATGA
