# Supplementary material for: Phage SPO1 Protein Gp49 Is a Novel RNA Binding Protein That Is Involved in Host Iron Metabolism
Source: Int J Mol Sci. 2023 Sep 20;24(18):14318. doi: 10.3390/ijms241814318 (PMC10531801; doi:10.3390/ijms241814318)
Supplement: Supplementary file 1 [file ijms-24-14318-s001.zip › Sup_tables1-3/Table S3.pdf]

Table S3: NMR statistics

**NMR Distance and Dihedral Constraints**	
Distance constraints	
Total NOE	1238
Intraresidue	670
Interresidue	568
Sequential (|*i*-*j*|)=1)	232
Short range (2≤|*i*-*j*|)≤3)	162
Medium range (4≤|*i*-*j*|)≤5)	76
Long range (|*i*-*j*|)>5)	98
Total Dihedral angle Restraints	310
φ	105
ψ	105
Total RDCs	0
**Structural Statistics**	
Violations (mean and SD)	
Distance constraints (Å)	0.023 ± 0.004
Dihedral angle constraints (°)	0.55 ± 0.091
Maximum dihedral angle violation (°)	0.84
Maximum distance constraint violation (Å)	0.32
Deviations from idealized geometry	
Bond length (Å)	0.0015 ± 0.000
Bond angle (°)	0.333 ± 0.005
Impropers (°)	0.284 ± 0.017
Average Pairwise rmsd (Å)	
Heavy	0.549 ± 0.0429
Backbone	0.411 ± 0.0750
